# Effects of Transcranial Direct Current Stimulation Paired With Cognitive Training on Functional Connectivity of the Working Memory Network in Older Adults

**DOI:** 10.3389/fnagi.2019.00340

**Published:** 2019-12-16

**Authors:** Nicole R. Nissim, Andrew O’Shea, Aprinda Indahlastari, Jessica N. Kraft, Olivia von Mering, Serkan Aksu, Eric Porges, Ronald Cohen, Adam J. Woods

**Affiliations:** ^1^Center for Cognitive Aging and Memory, Department of Clinical and Health Psychology, McKnight Brain Institute, University of Florida, Gainesville, FL, United States; ^2^Department of Neuroscience, University of Florida, Gainesville, FL, United States

**Keywords:** working memory, transcranial direct current stimulation, cognitive training, cognitive aging, functional connectivity, fMRI, N-Back, neuromodulation

## Abstract

**Background:**

Working memory, a fundamental short-term cognitive process, is known to decline with advanced age even in healthy older adults. Normal age-related declines in working memory can cause loss of independence and decreased quality of life. Cognitive training has shown some potential at enhancing certain cognitive processes, although, enhancements are variable. Transcranial direct current stimulation (tDCS), a form of non-invasive brain stimulation, has shown promise at enhancing working memory abilities, and may further the benefits from cognitive training interventions. However, the neural mechanisms underlying tDCS brain-based enhancements remain unknown.

**Objective/Hypothesis:**

Assess the effects of a 2-week intervention of active-tDCS vs. sham paired with cognitive training on functional connectivity of the working memory network during an N-Back working memory task.

**Methods:**

Healthy older adults (*N* = 28; mean age = 74 ± 7.3) completed 10-sessions of cognitive training paired with active or sham-tDCS. Functional connectivity was evaluated at baseline and post-intervention during an N-Back task (2-Back vs. 0-Back).

**Results:**

Active-tDCS vs. sham demonstrated a significant increase in connectivity between the left dorsolateral prefrontal cortex and right inferior parietal lobule at post-intervention during 2-Back. Target accuracy on 2-Back was significantly improved for active vs. sham at post-intervention.

**Conclusion:**

These results suggest pairing tDCS with cognitive training enhances functional connectivity and working memory performance in older adults, and thus may hold promise as a method for remediating age-related cognitive decline. Future studies evaluating optimal dose and long-term effects of tDCS on brain function will help to maximize potential clinical impacts of tDCS paired with cognitive training in older adults.

**Clinical Trial Registration:**

www.ClinicalTrials.gov, identifier NCT02137122.

## Introduction

Working memory is a frontal lobe mediated short-term memory process that plays a critical role in numerous aspects of everyday life (Baddeley and Hitch, 1974; Baddeley, 1992, 2000, 2003). Working memory enables decision-making, problem solving, planning, and reasoning abilities. Declines in working memory are commonly observed in older adults and, consequently, may cause significant negative impacts to activities of daily living and functional independence (Mograbi et al., 2014). Structural and functional changes of brain regions within the working memory network are known to occur with advanced age and have been associated with working memory decline (Cabeza et al., 2002; Nissim et al., 2017, 2019). Thus, there is a strong need to identify targeted interventions that can enhance working memory processes in older adults. Interventions involving cognitive training have shown some potential for enhancing cognitive processes in the trained domain(s). However, variability exists between training programs, the benefits derived from training, and the durability of gains over time (Ball et al., 2002; Rebok et al., 2014).

Non-invasive brain stimulation techniques, such as transcranial direct current stimulation (tDCS), has shown potential for enhancing cognitive training effects in older adults (Park et al., 2013; Richmond et al., 2014; Gill et al., 2015; Jones et al., 2015; Stephens and Berryhill, 2016). tDCS, a safe and painless form of stimulation, functions by applying a weak direct electrical current through electrodes placed on the scalp to stimulate underlying brain tissue (Nitsche and Paulus, 2000; Kuo and Nitsche, 2015; Pelletier and Cicchetti, 2015; Woods et al., 2016). Prior studies examining tDCS as an adjunctive tool paired with cognitive training have demonstrated significant behavioral enhancements in active vs. sham groups, supporting a paired intervention approach being more beneficial than cognitive training alone (Park et al., 2013; Jones et al., 2015; Stephens and Berryhill, 2016). Stephens and Berryhill (2016) demonstrated that older adults who received active-tDCS vs. sham (anode over F4, cathode over contralateral cheek) during working memory training experienced greater benefits on untrained assessment tasks post-training. Combining working memory training with tDCS has shown to extend and increase training gains (Park et al., 2013; Richmond et al., 2014; Jones et al., 2015; Stephens and Berryhill, 2016). The efficacy and transfer of tDCS paired with working memory training may be state dependent, with greater transfer occurring when training tasks are more difficult (Gill et al., 2015). Mounting evidence suggests that the simultaneous pairing of tDCS with cognitive training can increase learning and enhance cognition over cognitive training alone. Martin et al. (2014) provided evidence showing the importance of timing tDCS (online vs. offline) with cognitive training in healthy adults. Participants received active-tDCS over the left dorsolateral prefrontal cortex (DLPFC) (2 mA for 30 min; anode over left DLPFC, cathode over the right upper arm) immediately before (offline) and during (online) performance on a working memory N-Back cognitive training task (online and offline test conditions were separated by 1 month). Results showed a significant association between tDCS paired with cognitive training and improved skill acquisition on the cognitive training task over tDCS applied before the training task (Martin et al., 2014). Furthermore, evidence from Gill et al. (2015) has shown that pairing tDCS (2 mA for 20 min; anode over the left DLPFC, cathode over the right supraorbital region) during a more challenging vs. a less challenging working memory cognitive training task can enhance performance on subsequent working memory assessments. Participants in this study performed a cognitive task with greater working memory load vs. lower working memory load (3-Back vs. 1-Back version of the N-Back) while receiving active or sham stimulation. Active-tDCS when paired with 3-Back training demonstrated significant improvements in accuracy and reaction time performance on another working memory task, an adjusted Paced Auditory Serial Addition Task (A-PASAT) over sham-tDCS. This improvement was not seen in participants that received sham-tDCS during the 3-Back, active-tDCS during the 1-Back, or sham-tDCS during the 1-Back (Gill et al., 2015). Collectively, these findings indicate the greater potential of improving cognitive outcomes when tDCS is paired with cognitive training and the relevance of tasks’ cognitive demands during stimulation in relation to the effects of tDCS. These data support our rationale to utilize tDCS during an adaptive cognitive training program in older adults.

While tDCS paired with cognitive training has shown promise for enhancing working memory function in older adults, the underlying neural mechanisms of these effects are not yet well understood (Jones et al., 2015; Stephens and Berryhill, 2016). In a recent study, we demonstrated that tDCS delivered bilaterally to the frontal lobes (F3/F4) increases functional connectivity between left ventrolateral prefrontal cortex (VLPFC) and left DLPFC in older adults during performance on a working memory task (Nissim et al., 2019). However, these effects were only evident during active stimulation and not immediately after stimulation was stopped (i.e., after-effects). While there is a robust literature on the after-effects of tDCS, these data suggest that the stability and duration of tDCS effects on cognitive brain function may differ. Whether the temporal limitation of tDCS effects on functional connectivity in the working memory network differs between acute and repeated sessions of stimulation during cognitive performance is unknown. These recent data also demonstrated that tDCS impacts the working memory network in an effort/state-dependent fashion, with increased connectivity occurring only during more challenging working memory performance (2-Back vs. 0-Back). Whether state-dependent effects of tDCS persist with repeat training and stimulation session also remains unknown. To our knowledge, no study to date has investigated the impact of multiple sessions of tDCS paired with cognitive training on brain function of the working memory network during functional MRI (fMRI) N-Back task performance. Such data would be critical for better understanding the mechanistic influence of tDCS on the brain and crucial for future efforts to optimize the efficacy of pairing cognitive training with tDCS in older adults.

The current study examined the effects of a 2-week intervention of active-tDCS vs. sham combined with working memory and speed of processing cognitive training on functional connectivity of the working memory network in healthy older adults. Working memory and speed of processing both strongly relate to frontal lobe mediated cognitive function. Moreover, speed of processing is known to decrease with older age and hypothesized to partially contribute to age-related decline in working memory (Ebaid et al., 2017; Park and Festini, 2017). Combined working memory and speed of processing cognitive training was selected to optimize potential impact on working memory function. Participants were assessed during an fMRI N-Back task (2-Back vs. 0-Back) at baseline and after ten-sessions of intervention to determine the impact of stimulation on working memory brain connectivity. We hypothesized that the active vs. sham group would exhibit increased functional connectivity in brain regions related to the working memory network during task performance at post-intervention. We also hypothesized that the active vs. sham group would demonstrate significantly improved performance on the higher effort 2-Back vs. 0-Back task.

## Materials and Methods

This phase II clinical pilot study employed a randomized, triple-blinded (assessor, interventionist, participant) between-subjects design. This approach enabled examination of the effects of an intervention combining tDCS with cognitive training on functional connectivity of the working memory network in healthy older adults. The trial was preregistered in clinicaltrials.gov under NCT02137122.

### Participants

**FIGURE 1:**
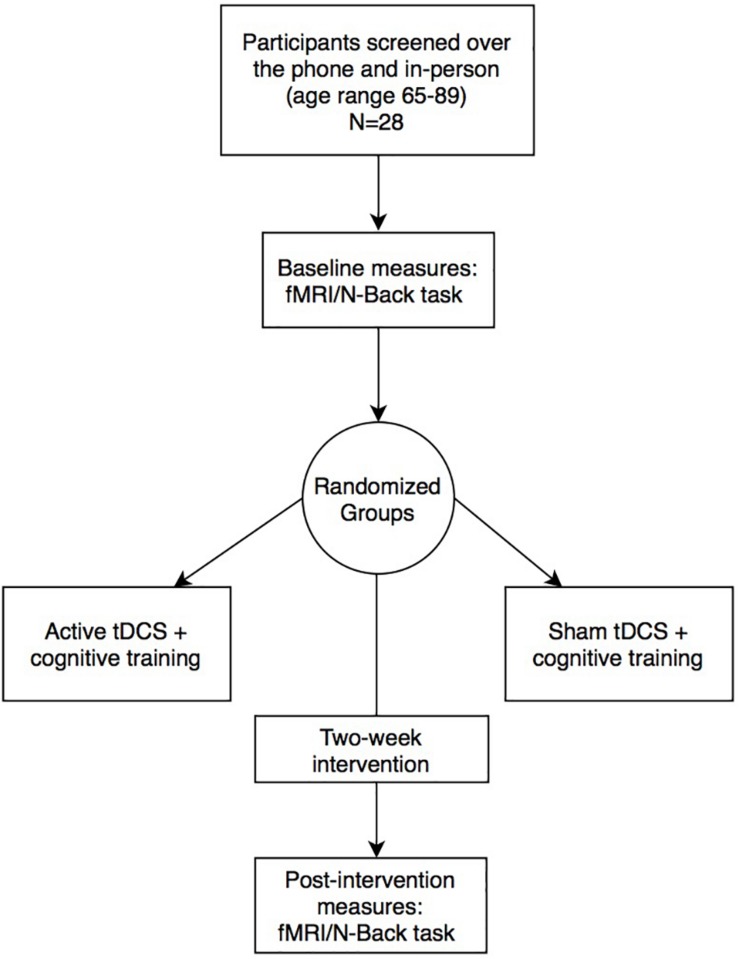
Experimental design and study timeline.

Twenty-eight healthy older adults (*n* = 14 active; *n* = 14 sham) were recruited in Gainesville and the surrounding North Florida areas. All participants underwent phone and in-person screening to verify eligibility based on the following study inclusion criteria: (1) Age 65–89 years old; (2) English speaking; (3) Physically mobile; (4) MRI compatible (no metal/other implants that contraindicate MRI); (5) Eligible for tDCS application on scalp; (6) No evidence of cognitive impairment as defined by the National Alzheimer’s Coordinating Center (NACC) Uniform Data Set (UDS-III) performance below 1.5 standard deviations on age/sex/education normative data in at least one cognitive domain (Woods et al., 2018); (7) Free from neurological or neurodegenerative disease and past opportunistic brain infection; (8) No loss of consciousness exceeding 20-min from traumatic brain injury; (9) Not taking medications that would impact tDCS effects (i.e., sodium channel blockers, glutamatergic or GABAergic medications) (McLaren et al., 2018); (10) Free from major psychiatric illness (schizophrenia, current substance dependence, severe major depression and/or suicidality); (11) Unstable and chronic medical conditions (e.g., cancer other than basal cell skin; severe uncontrolled diabetes); (12) Free of hearing or vision deficits that would impact ability to complete cognitive training or in-scanner tasks; (13) Right-hand dominant; (14) Score below 80% on the cognitive training POSIT assessment at the screening visit. The study protocol was in accordance with the Declaration of Helsinki and approved by the University of Florida’s Institutional Review Board. Informed written consent was obtained from participants prior to study procedures. Active vs. sham groups were not significantly different on age, sex, education, or Montreal Cognitive Assessment (MoCA) score (independent *t*-tests; *p* > 0.05). Figure 1 and Table 1 describes the study timeline and participant demographics, respectively.

**TABLE 1:** Demographics between stimulation groups and the total sample after preprocessing [mean, standard deviation (SD)].

**Group**	**Sample size**	**Age (SD)**	**Education (SD)**	**Sex**	**MoCA (SD)**
Active	14	73.57 (7.84)	17.00 (2.45)	7F:7M	27.85 (1.79)
Sham	14	73.78 (7.06)	17.42 (2.74)	8F:6M	27.00 (2.07)
Total	28	73.67 (7.32)	17.21 (2.42)	15F:13M	27.42 (1.95)

### tDCS Parameters and Application

**FIGURE 2:**
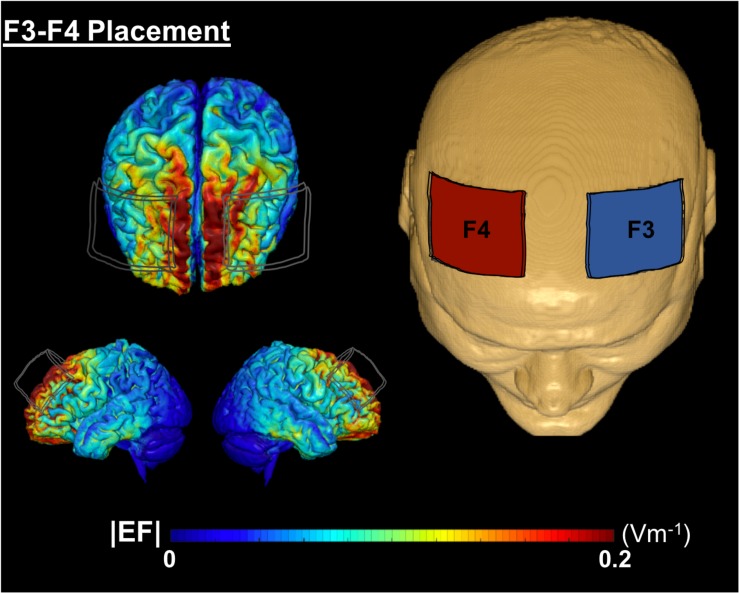
A computational model of electric field distribution for F3–F4 placement in one participant. The left hand side depicts the electric field strengths (|EF|) on the cortical surface for F3 (cathode, blue electrode) and F4 (anode, red electrode) montage. |EF| distribution was calculated using a finite element based approach in ROAST (Huang et al., 2019).

Conventional 1 × 1 tDCS (Soterix Medical, tDCS-CT for clinical trials) was applied at 2 mA intensity using two 5 × 7 cm^2^ saline-soaked Soterix sponge electrodes (0.9% NaCl; 4 mls per side, 8 ml total/sponge) at F3 (cathode) and F4 (anode) location, approximately over left and right DLPFC, respectively. A growing body of evidence demonstrates that 2 mA produces a net increase in excitability under the anode and cathode electrodes (Batsikadze et al., 2013; Mosayebi et al., 2019; Nissim et al., 2019). We chose this montage to elicit excitability under both the anode and cathode electrodes which is supported by prior behavioral and connectivity findings with tDCS using this same montage (Nissim et al., 2019; Soyata et al., 2019). Figure 2 demonstrates a computational model of expected current flow for the bilateral frontal montage that was used in this study. The device provided experimenter-blinding capability using a unique six-digit code. Active vs. sham groups received identical set up procedures. For each session, participants underwent head measurements using the International 10–20 system for electrode locations. Each session included a 40-min computerized cognitive training with stimulation delivered during the first 20-min in both active and sham conditions. The active group underwent stimulation at 2 mA intensity for 20-min with a 30-s current ramp up and down. The sham group underwent 2 mA stimulation for 30-s with 30-s ramp up and down. The sham condition provided the sensation of active stimulation yet the shortened duration did not produce a biological meaningful effect. Participants were given a stimulation sensation questionnaire before and after each stimulation session to rate typical sensations experienced from tDCS on a 0–10 scale (e.g., tingling, itching, burning, pain, fatigue, nervousness, headache, difficulty concentrating, mood change, change in vision/visual perception, visual sensation at the start/end of stimulation). Participants were also given a blinding questionnaire after 2-weeks of intervention visits [Q1: Which brain stimulation treatment condition do you believe you received? (Active, Sham/Placebo, Don’t know/Unsure); Q2: If you answered “Don’t know/Unsure” above, can you please provide your best (or random) guess of the treatment you received anyway?; Q3: On a scale of 0–10, how confident are you that you received (your selection)?]. Sensation and blinding data are reported in Results.

### Cognitive Training Procedure: POSIT Science BrainHQ

An adaptive, computerized cognitive training program was performed through POSIT Science BrainHQ^1^, which has been validated for producing significant cognitive and functional improvements in older adults (Berry et al., 2010). All participants in the study were instructed on how to perform the tasks at the screening visit in an identical manner. In each session, participants were randomly assigned to train on four out of eight adaptive tasks, totaling of 40-min of cognitive training per day (10-min/task). All participants received equal number of trainings across the eight tasks. The program included four working memory related tasks: card shark (visual N-Back task), auditory aces (auditory N-Back task), memory grid, and to-do-list; and four speed of processing tasks: double decision (useful field of view – UFOV), divided attention, hawk eye, and target tracker.

### Structural and Functional Neuroimaging Acquisition

Structural T1-weighted MPRAGE images and fMRI data were collected for each participant at baseline and post-intervention. Data was obtained on a 3-Tesla Siemens Prisma scanner using a 32-channel receive-only head coil. High-resolution T1-weighted images were acquired using the following protocol: repetition time (TR) = 1800 ms; echo time (TE) = 2.26 ms; flip angle = 8°; field of view (FOV) = 240 mm × 240 mm × 170 mm; voxel size = 1 mm^3^; scan duration = 183-s. Functional images were collected using an echo planar imaging (EPI) sequence (44 axial slices, no gap, TR = 3000 ms, TE = 30 ms, flip angle = 70°, FOV = 240 mm, voxel size = 3 mm^3^, scan duration = 11-min and 30-s. The fMRI task was presented on a screen that was made visible to participants lying inside the scanner.

### N-Back Paradigm

**FIGURE 3:**
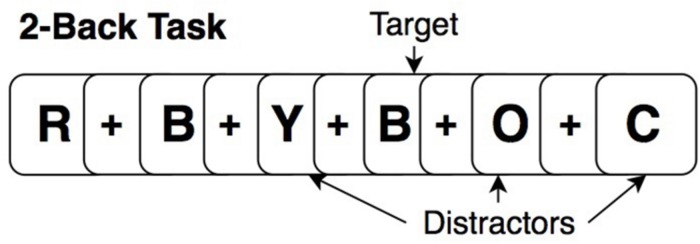
Example of a 2-Back working memory task.

At baseline and post-intervention, all participants performed an “untrained” (relative to cognitive training tasks) fMRI N-Back task (2-Back and 0-Back) in scanner, providing a measure of near transfer for training effects. The task paradigm for each run consisted of four blocks of 2-Back, four blocks of 0-Back in randomized order of presentation (15 stimuli per block; 5 targets; 10 distractors). Before each task block, a 20-s rest block occurred in which participants were instructed to focus on a dot in the center of the screen. This counted as the “rest” period. During the 2-Back task, participants viewed stimuli of uppercase letters one at a time on the screen with a crosshair (+) as the inter-stimulus interval between stimuli (Figure 3). Each stimulus appeared for 1-s, and the cross hair for 3-s, providing a 4 s window to respond. Participants were instructed to respond by button press with their right hand, using the index finger when the current letter matched the letter that appeared two-trials back (targets) and a different button with the middle finger when stimuli did not match the 2-Back pattern (distractors). The 0-Back was identical to the 2-Back in that it provided similar visual input and motor responses; however, it lacked the pattern component and was used as an attention-control task. For 0-Back, participants were instructed to press a button with the index finger only for the letter “X” and a different button with the middle finger for any other letter. All participants were trained to perform the N-Back out of the scanner identically, before the MRI, and were reminded of instructions while inside the scanner prior to starting the task.

### Neuroimaging Preprocessing

Spatial and functional imaging data were preprocessed through the CONN Toolbox default preprocessing pipeline (Whitfield-Gabrieli and Nieto-Castanon, 2012), which utilized some components from SPM12 (Friston et al., 2007) running on MATLAB version 2015R (The MathWorks Inc., United States). Preprocessing steps for each participant included T1 segmentation into gray matter, white matter, and cerebrospinal fluid, and normalization into Montreal Neurological Institute (MNI) space. The functional volumes underwent realignment, slice-timing correction, normalization to MNI space using normalized EPI templates in CONN, and a spatial Gaussian smoothing kernel of 8 mm. The artifact detection toolbox was applied to detect motion artifacts. The computed motion parameters were then used to remove outliers, which were the volumes with global signals exceeding thresholds of either 3 mm (translation) or 1° rotation. No volumes exceeded motion thresholds in either group.

### Functional Connectivity Processing

After preprocessing, data were denoised and filtered (0.008-infinity Hz) to reduce low-frequency drift and noise effects. Temporal filtering was used to remove effects from low and high frequency oscillations (scanner drift, head motion, heart rate, and respiration rate). The anatomical component-based noise correction (aCompCor) method within CONN was employed to perform noise correction (Behzadi et al., 2007). This method extracted principal components from the white matter and CSF time series, and utilized them as confounds during the denoising step (Behzadi et al., 2007; Whitfield-Gabrieli and Nieto-Castanon, 2012; Demirakca et al., 2016). This was done to reduce any physiological (or other noise source) and participant movement from the time series of interest, allowing for enhanced sensitivity, specificity, and validity for first- and second-level connectivity analyses (Whitfield-Gabrieli and Nieto-Castanon, 2012; Fallon et al., 2016). After denoising, first-level region of interest (ROI-to-ROI) analyses were performed to assess functional connectivity for each ROI (seed) to all other ROIs (targets) in the working memory network using a bivariate regression, generalized psychophysiological interaction (gPPI) approach for the 2-Back and 0-Back tasks. The N-Back blocks in each run were synchronized with the functional data to capture only the task period, removing resting and instructional periods. Fisher-transformed bivariate regression coefficients (connectivity β values) between two ROI BOLD time-series were used to demonstrate significant increases or decreases in functional connectivity between ROIs.

### Spherical Region of Interests Selection

Spherical ROIs involved in working memory were generated using the WFU PickAtlas GUI through SPM12. Previously, the Owen et al. (2005) meta-analysis identified significant coordinates of BOLD activation during N-Back working memory tasks from 24 fMRI studies. Fifteen ROIs in the Owen et al. (2005) meta-analysis were selected *a priori* to represent the working memory network. The ROIs were chosen for specific activation in verbal working memory identity monitoring N-Back paradigms. Each ROI was created in MNI space (transformed from Talairach) using the peak coordinate and volume (mm^3^, approximated to a spherical shape) as reported in the meta-analysis (Table 2). For any ROI without an associated volume, the default 10 mm diameter was used.

**TABLE 2:** MNI coordinates for each ROI and radius of sphere.

**Region**	***x***	***y***	***z***	**Radius**
LH DLPFC	–37.75	50.19	13.6	6.2
	–46.26	22.71	18.6	14.3
LH Frontal pole	–37.75	50.19	13.6	7.5
LH Inferior parietal lobule	–37.09	–47.7	45.58	10
LH Lateral premotor	–26.32	6.75	53.46	9
	–45.96	3.1	38.47	10
LH Ventrolateral PFC	–31.36	21.11	0.58	10
Medial cerebellum	3.12	–69.09	–24.69	3
RH DLPFC	44.53	38.76	24.43	12.5
RH IPL	44.97	–45.49	41.73	12.46
RH lateral premotor	31.96	11.01	49.8	15.83
	31.96	11.01	49.8	10
RH Medial posterior parietal	12.77	–63.71	55.28	14.8
RH Ventrolateral PFC	35.58	23.26	–3.01	10
Supplementary motor area	–0.588	18.57	40.65	10

*LH: left hemisphere; RH: right hemisphere.*

### Statistical Analyses

#### Functional Connectivity

Second-level group analyses were performed in CONN to identify the impact of active vs. sham stimulation on functional connectivity of the working memory network during N-Back task performance from baseline to post-intervention. The second-level data were modeled as a 2 × 2 design [Group (active vs. sham) by Time (baseline vs. post-intervention)]. The interaction between Group × Time was assessed to determine treatment effects. Change in functional connectivity within the working memory network (ROI-to-ROI, Table 2) from baseline to post-intervention was compared for active vs. sham group on 2-Back and 0-Back. Each ROI was assessed separately to identify potential changes in functional connectivity from baseline to post-intervention. Control analyses were then performed to evaluate whether active vs. sham group differed at baseline for either 2-Back or 0-Back connectivity (ROI-to-ROI), and modeled at the second-level as a 2 (Group) × 1 (Baseline) design. In all contrasts, age and education were used as covariates. To control for multiple comparisons, *p*-FDR correction of 0.05 was applied to all analyses to identify significant connectivity findings. Table 3 summarizes all assessed contrasts and rationales for examination.

**TABLE 3:** Description and rationale of second level contrasts.

**Between-subject effects**		**Between conditions**	**Rationale**
Active vs. sham	Primary contrasts	2-Back: Post-intervention > Baseline	Enabled assessment of connectivity changes on the 2-Back from baseline to post-intervention
		0-Back: Post-intervention > Baseline	Enabled assessment of changes in connectivity from baseline to post-intervention on the 0-Back task
	Control contrasts	2-Back: Baseline > Rest	Control contrast to determine if baseline differences existed on 2-Back between groups
		0-Back: Baseline > Rest	Control contrast to determine if baseline differences existed on the 0-Back between groups

#### N-Back Behavioral Performance

Percent accuracy for each component (targets, distractors, and the total average) and reaction time were analyzed separately for 2-Back and 0-back in IBM SPSS V25. Reaction time was obtained in milliseconds and log-transformed to achieve a normal distribution; mean reaction time was taken for targets and distractors on the 2-Back and 0-Back tasks. Repeated measures ANOVA was used to evaluate performance, with time as the within-subject factor (two levels: baseline and post-intervention) and stimulation condition as the between-subject factor. Task percent accuracy (i.e., 2-Back targets) or reaction time was the dependent variables. Age and education were included as covariates.

#### Sensation and Blinding Questionnaires

Sensation questionnaire data were averaged across 10-sessions for each participant for ratings given before, during and after stimulation. Mean ratings were analyzed in SPSS for each of the 11 categories using independent samples *t*-tests to evaluate the skin sensation/experience separately for before, during and after stimulation between treatment groups (Active vs. Sham). Blinding questionnaire data were analyzed using chi-square tests for Q1 and Q2 and an independent samples *t*-test for Q3 to evaluate blinding efficacy.

## Results

### Effects of Intervention on Functional Connectivity in the Working Memory Network

#### 2-Back: Post-intervention > Baseline

**FIGURE 4:**
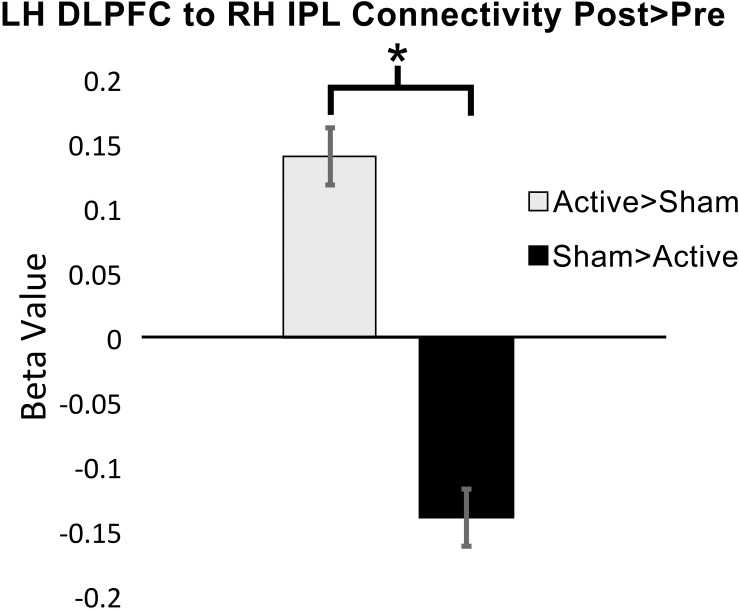
Mean functional connectivity β values (±standard error) for 2-Back over rest at baseline and post-intervention from left dorsolateral prefrontal cortex (DLPFC) to right inferior parietal cortex (IPL), ^∗^*p*-FDR < 0.05 (LH = left hemisphere; RH = right hemisphere).

**FIGURE 5:**
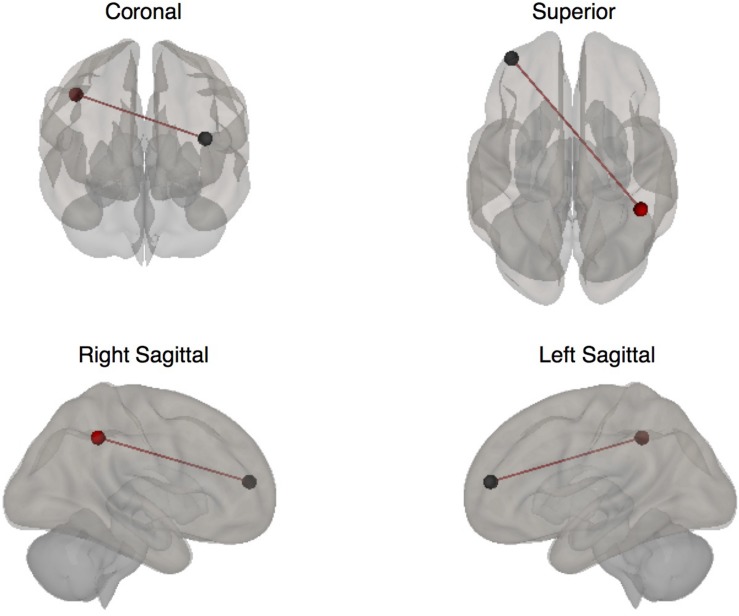
Seed to target ROIs with significantly increased connectivity during the 2-Back task at post-intervention (ROI colors: Red = left DLPFC; Black = right IPL).

Functional connectivity was significantly increased at post-intervention for active vs. sham on the 2-Back task between the left DLPFC to the right inferior parietal lobule (IPL): β = 0.14; *p*-FDR = 0.009; Hedge’s *G* = 1.40. Figure 4 demonstrates pattern of change in mean β values for 2-Back connectivity from left DLPFC to right IPL from baseline to post-intervention. Figure 5 depicts the significant ROI-to-ROI location. No other connections were found to be significant.

#### 0-Back: Post-intervention > Baseline

No significant connectivity changes were observed between any ROIs (*p*-FDR > 0.05).

### Evaluation for Baseline Differences in Functional Connectivity Between Groups

#### 2-Back: Baseline > Rest

A significant decrease in connectivity was observed when examining the supplementary motor area (SMA) ROI to the right lateral premotor cortex ROI during 2-Back task performance at baseline over rest (β = −0.17; *p*-FDR = 0.027; Hedge’s G = 1.23). However, no additional significant changes in connectivity were observed for any other ROI-to-ROI including the regions that demonstrated increased connectivity on 2-Back post-intervention over baseline.

#### 0-Back: Baseline > Rest

No significant changes in connectivity were observed between any ROIs (*p*-FDR > 0.05).

### Effects of Intervention on N-Back Behavioral Performance

#### Accuracy and Reaction Time

**FIGURE 6:**
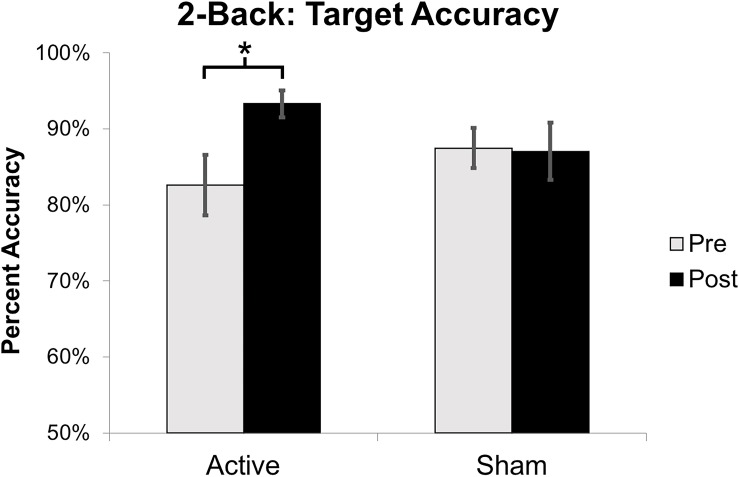
Mean percent accuracy for 2-Back target stimuli. Error bars represent standard error of the mean (^∗^*p* < 0.05).

**FIGURE 7:**
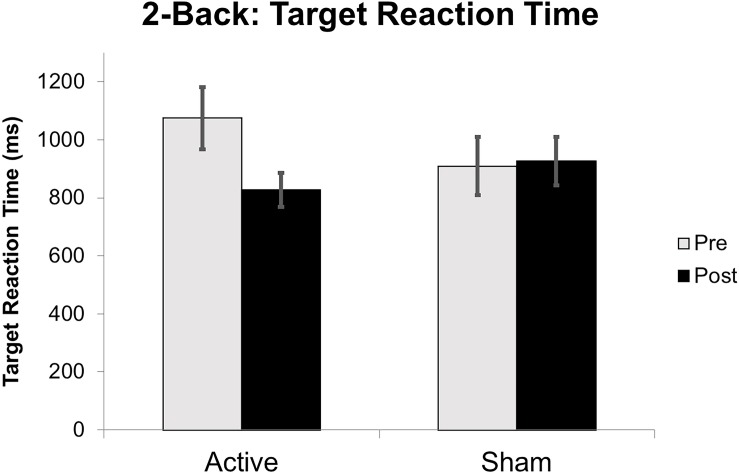
Reaction time on 2-Back target stimuli reported in milliseconds (ms) from baseline to post-intervention. Error bars represent standard error of the mean.

Target accuracy on the 2-Back task significantly improved at post-intervention in the active vs. sham group (time by stimulation condition; *F* = 6.226 df = 1.0; *p* = 0.020; partial eta squared = 0.206; observed power = 0.668); Figure 6. We assessed baseline 2-Back behavior and found no significant differences between the active vs. sham group (*p* > 0.05). Evaluation of linear and non-linear fits for 2-Back target reaction time demonstrated a non-significant trend toward faster responses (by 246.38 ms) in active vs. sham group (time by stimulation condition; *F* = 2.553; df = 1.0; *p* = 0.123; partial eta squared = 0.096; observed power = 0.335); Figure 7. Distractor accuracy and reaction time on the 2-Back task did not significantly differ between groups (*p* > 0.05). Performance accuracy and reaction time on 0-Back did not significantly differ between groups (*p* > 0.05).

### Sensation Ratings and Blinding Efficacy

No significant differences were observed in sensation ratings (Table 4) before, during or after stimulation between the active vs. sham group (*p*’s > 0.05). Chi-squared analyses between groups demonstrated that participants did not significantly endorse either Active, Sham or Unsure categories at a greater frequency between groups on Q1 (χ^2^ = 2.01, *p* = 0.36). For those answering Unsure, when forced to choose either Active or Sham for Q2, neither group significantly endorsed either choice more than the other group (χ^2^ = 3.23, *p* = 0.07). When participants rated their confidence in choice of active or sham, neither group significantly differed in their level of confidence (*t* = 0.261, *p* = 0.79). Of potential importance, it should be noted that changes in mood, as assessed by the Mood Change question, were not significantly different between active vs. sham groups at any timepoint (before, during and after stimulation). This suggests that effects on cognition were not related to changes in mood.

**TABLE 4:** Sensation rating (0–10 scale) before, during, and after stimulation.

	**Before stimulation**						**During stimulation**						**After stimulation**					
	**Active**		**Sham**				**Active**		**Sham**				**Active**		**Sham**			
**Ratings**	***M***	**SD**	***M***	**SD**	***t***	***p***	***M***	**SD**	***M***	**SD**	***t***	***p***	***M***	**SD**	***M***	**SD**	***t***	***p***
Tingling	0.04	0.13	0.08	0.23	–0.97	0.33	0.90	1.25	1.33	1.08	–0.67	0.51	0.37	1.14	0.25	0.37	0.23	0.72
Itching	0.37	1.18	0.08	0.22	1.23	0.22	0.60	1.38	0.13	0.32	0.90	0.37	0.57	1.35	0.63	1.06	–0.15	0.88
Burning	0.30	1.09	0.10	0.23	–0.004	0.99	1.28	2.06	1.28	1.53	0.66	0.51	0.47	1.36	0.54	0.96	–0.18	0.86
Pain	0.15	0.53	0.49	1.22	0.04	0.96	0.56	1.55	0.53	1.22	–0.96	0.34	0.59	1.65	1.53	2.47	–1.18	0.25
Fatigue	0.50	1.18	0.64	0.94	–0.83	0.41	0.39	1.01	0.71	1.00	–0.34	0.73	0.50	0.96	1.00	1.13	–1.25	0.21
Nervousness	0.09	0.19	0.12	0.37	–1.19	0.24	0.12	0.24	0.33	0.63	–0.24	0.81	0.07	0.15	0.23	0.49	–1.19	0.24
Headache	0.20	0.59	0.13	0.30	0.41	0.68	0.19	0.56	0.12	0.27	0.38	0.71	0.21	0.61	0.45	0.60	–1.06	0.29
Difficulty concentrating	0.39	0.86	0.42	0.64	–1.69	0.10	0.51	0.87	1.05	0.83	–0.11	0.91	0.28	0.64	0.61	0.64	–1.37	0.18
Mood change	0.19	0.40	0.21	0.39	–0.80	0.43	0.14	0.31	0.24	0.31	–0.09	0.92	0.14	0.43	0.36	0.55	–1.15	0.25
Vision change	0.04	0.08	0.03	0.09	–0.23	0.81	0.07	0.12	0.09	0.17	0.16	0.87	0.12	0.27	0.07	0.16	0.49	0.62
Visual sensation (phosphenes)	0.00	0.00	0.00	0.00	1.36	0.18	0.02	0.06	0.00	0.00	0.32	0.33	0.04	0.07	0.01	0.05	1.08	0.29

**M* = mean, SD = standard deviation, *t* = independent *t*-test, *p* = *p*-value.*

## Discussion

The main findings of this study demonstrate that active-tDCS paired with cognitive training selectively impacts functional connectivity of regions involved in the working memory network when compared to sham-tDCS paired with cognitive training in older adults. The significant improvement on 2-Back target accuracy in the active vs. sham group indicates that tDCS is capable of enhancing working memory performance beyond cognitive training alone (sham tDCS paired with cognitive training). The lack of change in functional connectivity or behavioral performance between groups on the less challenging 0-Back task suggests that tDCS-induced enhancements may occur in an effort/state-dependent manner.

It is important to understand what an increase in connectivity between the DLPFC and IPL might indicate for working memory processing in older adults. In terms of individual roles of functional regions within the working memory network, it is difficult to disentangle the distinct role of each region, as there can be an overlap in functional activation. Some regions have shown specificity for sensory modality of stimuli, whereas others, including parts of the parietal cortex and the DLPFC, have demonstrated to be active across multiple modalities (Klingberg et al., 1995; Curtis and D’Esposito, 2003; Linden, 2007; Klingberg, 2010). Thus, the connectivity increase between DLPFC and IPL might reflect a more fundamental and/or multi-modal role in processing of working memory. Functional neuroimaging studies in humans have previously confirmed the involvement of frontal and parietal cortices in working memory, in addition to their co-activation during working memory task performance (Owen et al., 2005). Prefrontal cortex and intra-parietal brain activation has been shown to correlate with working memory capacity differences in adults (Manoach et al., 1997; Edin et al., 2009). Previous research suggests a strengthened fronto-parietal connection as one possible mechanism supporting working memory capacity (Edin et al., 2009) with evidence to support the integral role of the frontal/DLPFC and parietal lobes in working memory processes (e.g., capacity and working memory load). Our results of increased connectivity between the left DLPFC and right IPL suggest tDCS may enable greater coherence between these regions and underlie behavioral improvement in working memory performance.

The current study identified significantly enhanced 2-Back accuracy in active vs. sham stimulation at post-intervention. This finding lends support to prior behavioral studies that have shown active-tDCS enhances N-Back working memory performance over sham (Zaehle et al., 2011). Prior research has also indicated the level of difficulty or effortfulness of the cognitive task during tDCS may impact behavioral outcomes. Gill et al. (2015) demonstrated significantly improved accuracy and reaction time on a working memory related task only after participants performed a more challenging task (3-Back vs. 1-Back) during a single session of active-tDCS. No improvements in behavior were observed after sham stimulation on either task difficulty level. Nissim et al. (2019) identified significantly increased functional connectivity between two frontal working memory ROIs during an acute single session of active stimulation while participants performed a 2-Back task (Nissim et al., 2019). Both the prior study and the current study only demonstrated significant changes in connectivity during the more challenging 2-Back and not the less challenging 0-Back task with active stimulation. The current study also demonstrated significantly increased target accuracy for the active group only on the more challenging 2-Back task. Collectively, these findings suggest cognitive demands of the task during tDCS influences the response and effects from active stimulation on behavioral performance and brain-based neural effects (Gill et al., 2015; Nissim et al., 2019).

Dose plays a major role in tDCS response and after-effects, thus, repeated tDCS sessions might prolong stimulation effects (Monte-Silva et al., 2013). tDCS applications on motor cortex have shown that repeated stimulation sessions in a specific time window can induce a stable late-phase long term potentiation (LTP) in humans (Monte-Silva et al., 2013). While this study did not aim to assess varying doses or frequency of sessions, we found differences in the spread of functional connectivity changes from active-tDCS vs. sham when comparing our current results to prior results involving a single acute session of tDCS during an fMRI N-Back task. We previously examined an acute single session of active-tDCS vs. sham over bilateral DLPFC (F3/F4, cathode/anode) at 2 mA during an fMRI N-Back task (2-Back vs. 0-Back) in older adults. Acute active-tDCS demonstrated significant increased connectivity during active stimulation between the left DLPFC to left VLPFC during the 2-Back. Sham stimulation did not result in connectivity changes during the 2-Back. No differences in connectivity were observed on the 0-Back task between groups. These data suggest that acute tDCS may produce more focal connectivity changes as the indicated increase was lateralized to the left frontal lobe during 2-Back task performance (Nissim et al., 2019). In contrast, the current study involved ten-repeated stimulation sessions in the active group and identified more distal ROIs that increased connectivity, from the left frontal lobe to the right parietal lobe. These results may reflect a broader spread of coherence in major hubs within the working memory network associated with repeated stimulation sessions; thus, provide support for the ability of tDCS to modulate functional connectivity. Moreover, comparing the single acute tDCS study vs. repeated sessions of tDCS suggest that increasing sessions can produce more distributed neural effects within the working memory network. Increased connectivity from the left to right hemisphere provides supporting evidence for mechanistically different processes that might be occurring with repeated stimulation vs. acute tDCS. This broader increased connectivity pattern could relate to timing mechanisms that aid in producing the late-phase LTP and long-term after-effects from tDCS that have been demonstrated in human tDCS studies of the motor cortex (Monte-Silva et al., 2013). The importance of intensity should also be considered in relation to the observed increased connectivity effects from active vs. sham stimulation as observed in the current study. It has previously been shown in motor cortex studies that 2 mA intensity increases the net excitability of motor evoked potentials (MEPs) under both the anode and cathode electrodes, whereas 1 mA increases excitability under the anode but decreases excitability under the cathode electrode (Batsikadze et al., 2013). This study aimed for net excitability increases under both the anode and cathode electrodes to provide broad net excitation to the left and right frontal lobes. We relied on the assumption that excitability effects in the frontal lobes behave similarly to motor cortex; our results provide evidence to support prior intensity findings through demonstrating that 2 mA active-tDCS increased functional connectivity between the left frontal to right parietal lobe. However, the role of intensity and its impact on brain-based effects from tDCS needs further clarification to determine optimal dose for enhancing cognitive processes.

## Limitations

The current study has several limitations that are important to address. The sample size was relatively small, which limited the potential detection of more subtle brain-based effects. However, this was a pilot study, and despite being potentially underpowered, results demonstrated significantly increased functional connectivity of brain regions involved in working memory and improved 2-Back target task accuracy. In addition, the effect sizes of assessments used in this study demonstrate medium to large effects. Nonetheless, larger intervention studies are warranted (Woods et al., 2018).

The cognitive training program used in this study targeted both speed of processing and working memory. While this was specifically chosen to impart the greatest benefit of executive functioning skills, targeting multiple domains may have limited the effects on working memory enhancements. It is possible that training focused specifically on working memory tasks could provide greater transfer to untrained assessments of working memory. Future studies designed to assess the effects of single modality training would better enable an understanding of single modality vs. multiple modality training transfer with tDCS.

This study assessed an intervention in older adults and did not include a young adult control group. Our results are limited to interpretation within a healthy older adult population. It is not currently known how working memory performance and functional connectivity might be modulated by tDCS paired with cognitive training in young adults. It is possible this approach may provide no benefit to young adults, as the working memory system has not yet experienced normal age-related decline and frontal lobe structures involved in working memory have not lost integrity in the young adult brain compared to older adults. Future studies assessing young vs. older adults on a paired intervention approach would better enable an understanding of whether repeated sessions of tDCS and cognitive training might impact functional connectivity in the young adult brain. Moreover, further research is required to evaluate whether patient populations with compromised working memory abilities demonstrate the same effects (e.g., mild cognitive impairment).

## Conclusion

Results from this study suggest that active-tDCS paired with cognitive training facilitates improvements in working memory network connectivity and behavioral gains on an untrained working memory task. Collectively, these data support the potential of tDCS to enhance working memory processes in healthy older adults and improve the impact of cognitive training interventions. Future research is needed to elucidate tDCS effects on brain function in a larger sample, optimize dose, and determine how long-term functional impacts from tDCS might alter working memory processing.

## Data Availability Statement

The datasets generated for this study are available on request to the corresponding author.

## Ethics Statement

The studies involving human participants were reviewed and approved by the University of Florida Institutional Review Board (IRB). The patients/participants provided their written informed consent to participate in this study.

## Author Contributions

All authors made substantial contributions to the conception and design of the study, acquisition of the data, analysis, interpretation of the data, involved in drafting and revising of the manuscript, and final approval of the submitted version.

## Conflict of Interest

The authors declare that the research was conducted in the absence of any commercial or financial relationships that could be construed as a potential conflict of interest.

## Footnotes

1 www.brainhq.com
